# Supplementary material for: Surface tension-assisted additive manufacturing
Source: Nat Commun. 2018 Mar 22;9:1184. doi: 10.1038/s41467-018-03391-w (PMC5864961; doi:10.1038/s41467-018-03391-w)
Supplement: Supplementary file 1 — Supplementary Information(PDF 2456 kb) [file 41467_2018_3391_MOESM1_ESM.pdf]

Supplementary Information

Surface tension-assisted additive manufacturing

Héloïse Ragelle,[1,2,3] Mark W. Tibbitt,[1,3,‡] Shang-Yun Wu,[1], Machael A. Castillo,[1] George Z. Cheng,[4] Sidharta P. Gangadharan,[5] Daniel G. Anderson,[1,3,6] Michael J. Cima,[1,7] and Robert Langer[*,1,6]

[1]The David H. Koch Institute for Integrative Cancer Research, Massachusetts Institute of Technology, 500 Main St Cambridge, MA 02142 USA
[2]Department of Anesthesiology, Boston Children's Hospital, Harvard Medical School, 300 Longwood Ave Boston, MA 02115 USA
[3]these authors contributed equally to this work
[4]Pulmonary, Allergy, and Critical Care Medicine, Department of Medicine, Duke University School of Medicine, 20 Duke Medicine Circle Durham, NC 27710 USA
[5]Department of Surgery, Beth Israel Deaconess Medical Center, Harvard Medical School, 330 Brookline Ave Boston, MA 02215 USA
[6]Department of Chemical Engineering, and Division of Health Science and Technology, Massachusetts Institute of Technology, 500 Main St Cambridge, MA 02142 USA
[7]Department of Materials Science and Engineering, Massachusetts Institute of Technology, 500 Main St Cambridge, MA 02142 USA
[‡]Current address: Macromolecular Engineering Laboratory, Department of Mechanical and Process Engineering, ETH Zürich, Sonneggstrasse 3, 8092 Zürich, Switzerland

*Corresponding author: rlanger@mit.edu.

Table of contents:

S. Figure 1	Force-displacement curves for uncoated tubular scaffolds	3
S. Figure 2	Force-displacement curves for hydrogel-coated tubular scaffolds	3
S. Figure 3	Repeat stress-strain measurements of a hydrogel-coated, anisotropic device	4
S. Figure 4	Schematic of the model geometry	5
S. Figure 5	Schematic of the geometry considered for Phase I filling	6
S. Figure 6	Schematic of the geometry considered for Phase II filling	6
S. Figure 7	Reduced Gibbs Energy as a function of surface energy	7
S. Figure 8	Reduced Gibbs Energy as a function of saturation	7
S. Figure 9	Reduced Gibbs Energy as a function of contact angle	8
S. Figure 10	Surface tension-assisted coating of a polycaptrolactone (PCL) scaffold	8
S. Table 1	Design parameters of the 3D printed scaffolds	9
S. Methods	Modeling of suspended liquid films	10
S. Discussion		18
S. References		20

Supplementary Figures

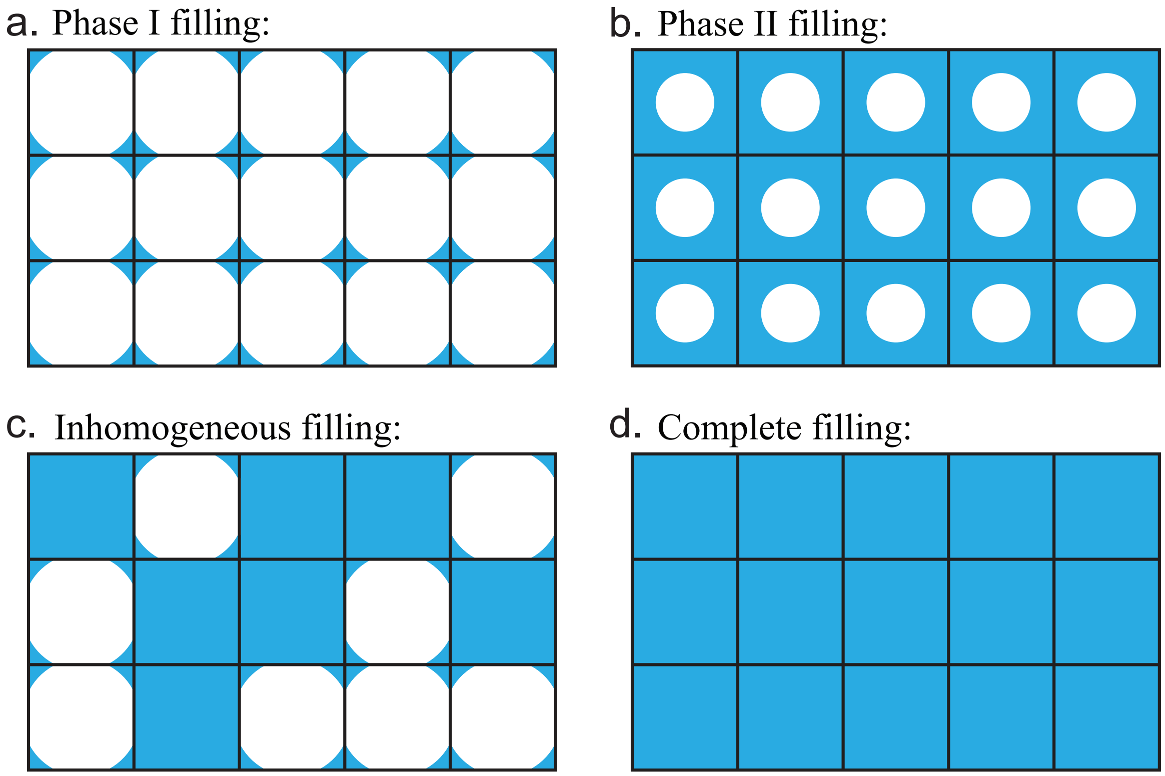

Supplementary Figure 1: Schematic of the geometry considered in the physical model of suspended liquid film formation on a two-dimensional, square-cell mesh. **a.** Phase I filling corresponds to liquid wetting in the corners or fillets of the square cells. The angle subtended by the fillet is controlled by the contact angle, θ. Phase I filling occurs only at low saturation. **b.** Phase II filling corresponds to the proposed scenario where further addition of liquid causes the fillets to meet allowing the cells to fill uniformly across the mesh with a single open circle in the center of each cell. Phase II filling is not observed physically as described below. **c.** Instead of Phase II filling, a real mesh will undergo Inhomogeneous filling at relatively low saturation where individual wells will be either completely filled or remain with liquid only in the fillets. **d.** Fortunately, for the purpose of this work, the completely filled cells are energetically favorable and further addition of liquid leads to Complete filling of the mesh, which is necessary for the surface tension-assisted additive manufacturing described here. This is true for a range of contact angles, $0° \leq \theta < 90°$, as described below, and offers a wide range of operating conditions for complete coating of reticulated scaffolds.

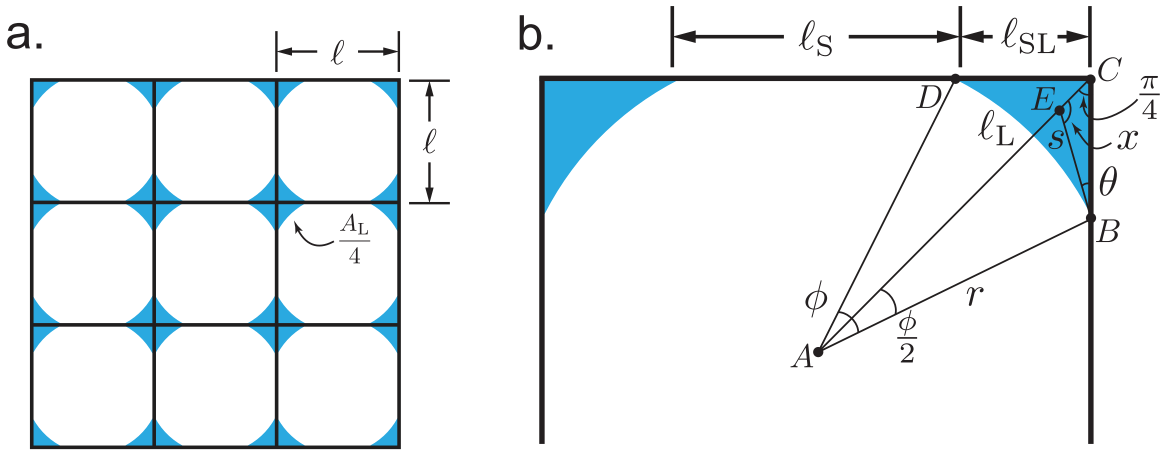

Supplementary Figure 2: Schematic of the geometry considered for Phase I filling. **a.** An overview of the two-dimensional, square-cell mesh with cell dimension, ℓ, and liquid area of a single fillet, $\frac{A_L}{4}$. **b.** An inspection of the geometry of a single corner during Phase I filling used to calculate the Gibbs Energy, F_I.

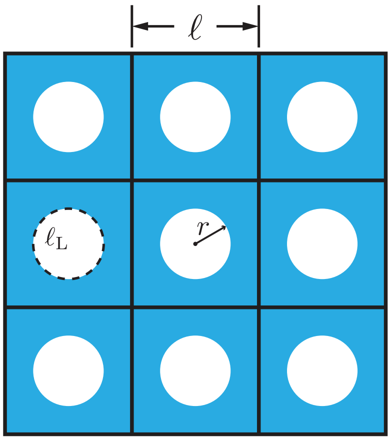

Supplementary Figure 3: Schematic of the geometry considered for Phase II filling with cell dimension, ℓ, and a single circle with radius, r.

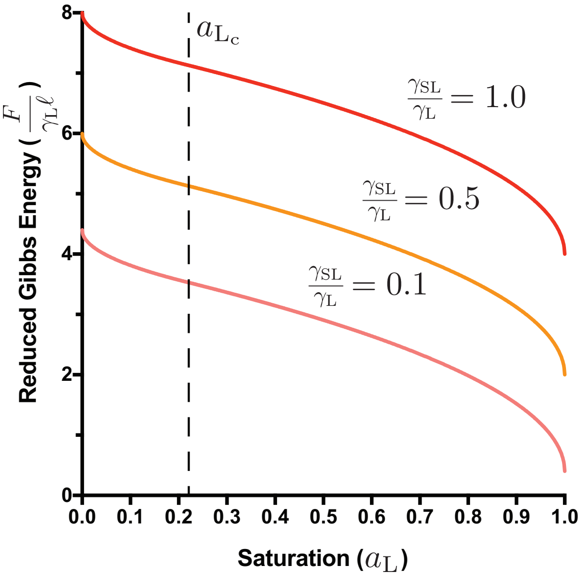

Supplementary Figure 4: A plot of the reduced Gibbs Energy, $\frac{F}{\gamma_{\mathrm{L}}\ell}$, as a function of saturation, a_{L}, for $\theta = 0°$ and $\frac{\gamma_{\mathrm{SL}}}{\gamma_{\mathrm{L}}} = 0.1$, 0.5, and 1.0.

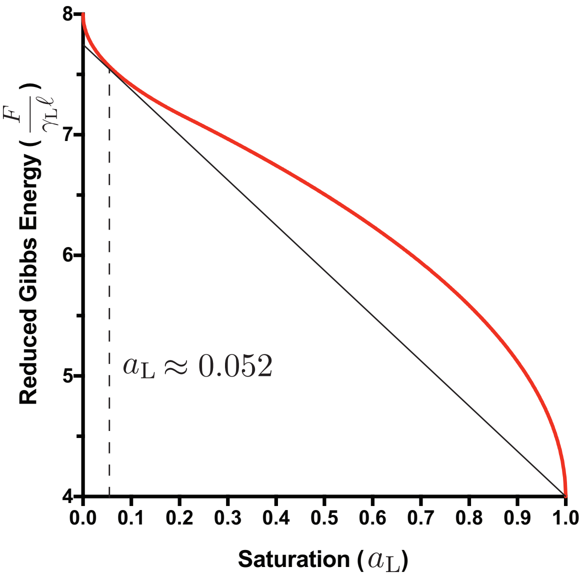

Supplementary Figure 5: A plot of the reduced Gibbs Energy, $\frac{F}{\gamma_{\mathrm{L}}\ell}$, as a function of saturation, a_{L}, for $\theta = 0°$ and $\frac{\gamma_{\mathrm{SL}}}{\gamma_{\mathrm{L}}} = 1.0$.

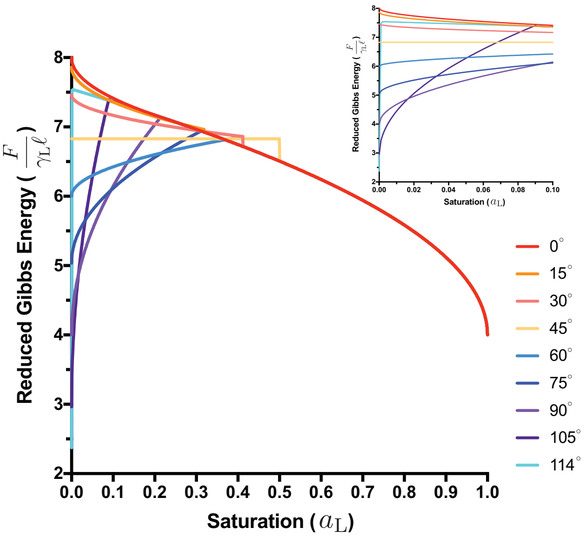

Supplementary Figure 6: A plot of the reduced Gibbs Energy, $\frac{F}{\gamma_L \ell}$, as a function of saturation, a_L, for θ = 0°, 15°, 30°, 45°, 60°, 75°, 90°, 105°, and 114° and $\frac{\gamma_{SL}}{\gamma_L}$ = 1.0. Inset graph with a_L = [0, 0.1]

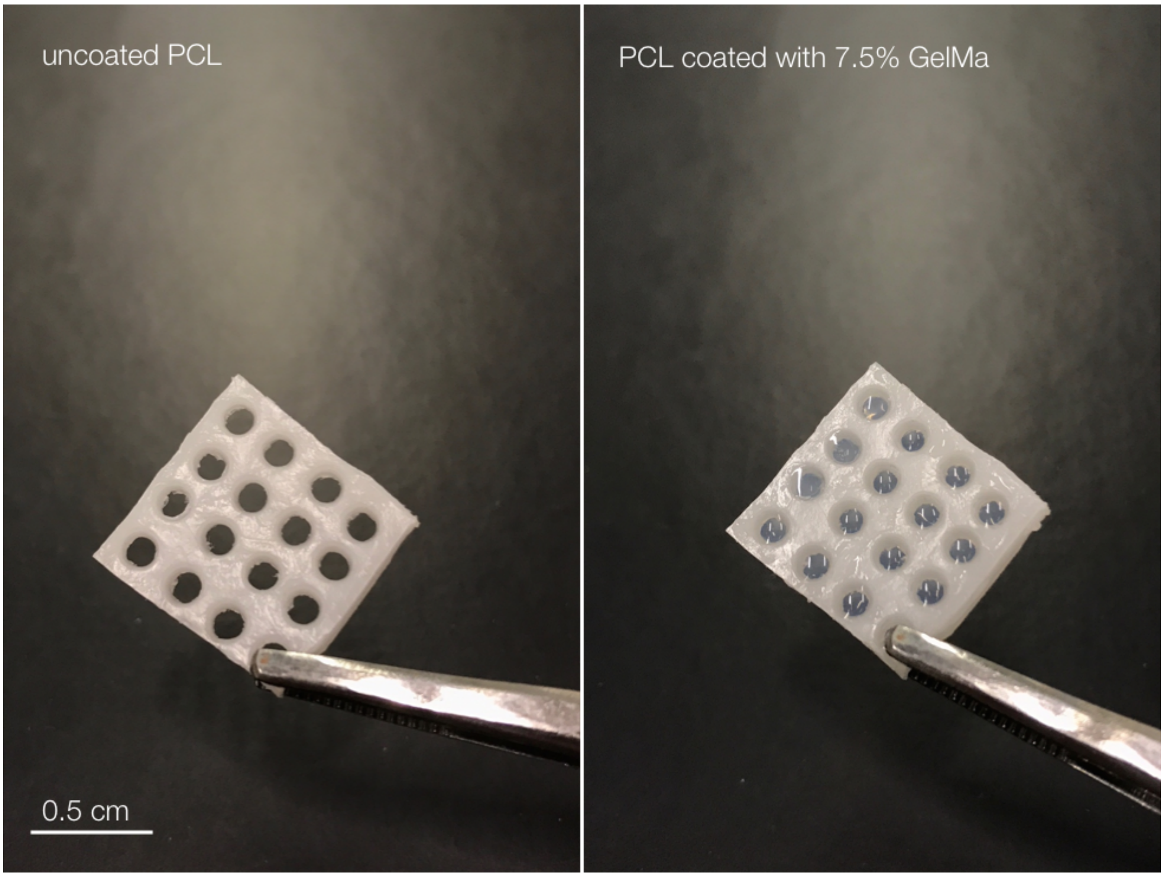

Supplementary Figure 7: Surface tension-assisted coating of a polycaprolactone (PCL) scaffold. **a.** A fenestrated PCL scaffold was fabricated as a biodegradable support for hydrogel coating. **b.** The PCL scaffold was successfully coated with methacrylated-gelatin as described for other materials.

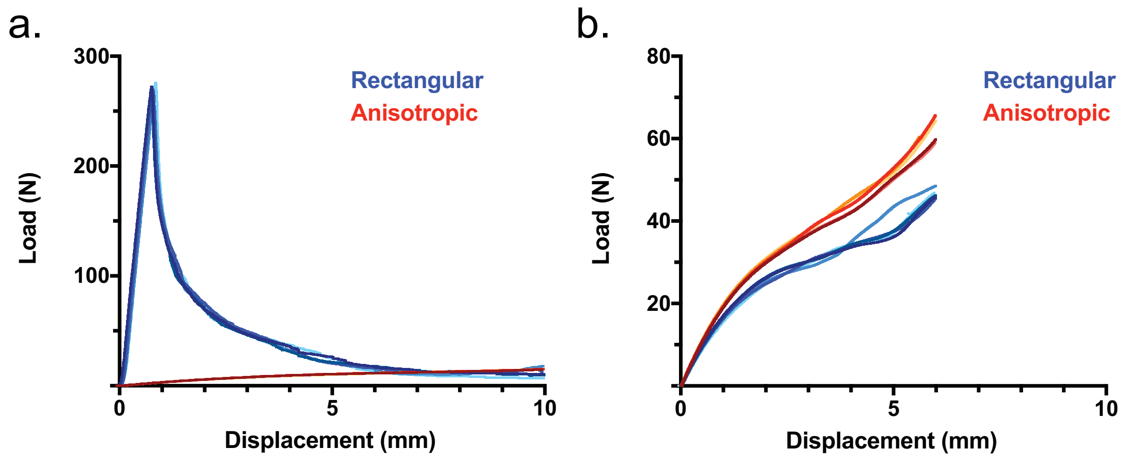

Supplementary Figure 8: Force-displacement curves for all of the uncoated tubular scaffolds. **a.** Compression tests conducted along the longitudinal axis. **b.** Compression tests conducted along the radial axis. n = 5.

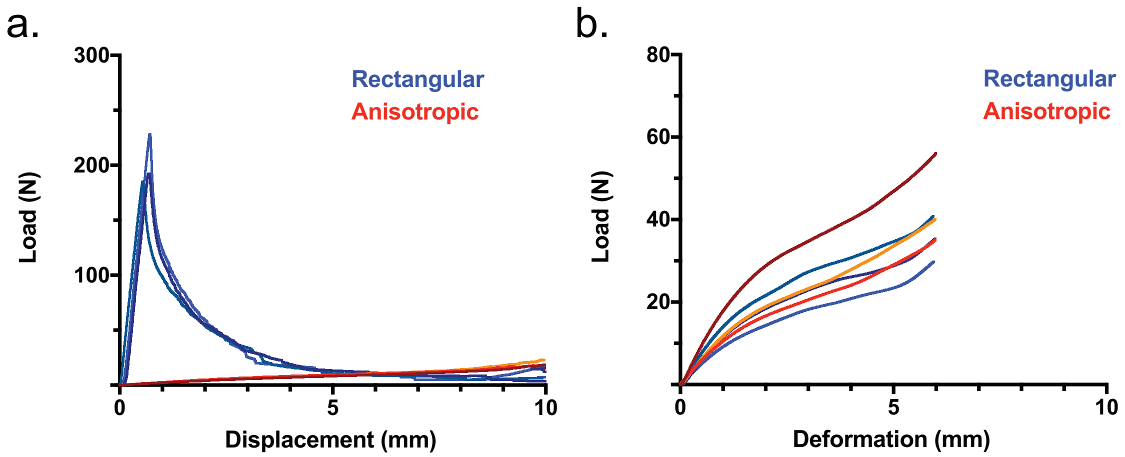

Supplementary Figure 9: Force-displacement curves for all of the hydrogel-coated tubular scaffolds. **a.** Compression tests conducted along the longitudinal axis. **b.** Compression tests conducted along the radial axis. n = 3.

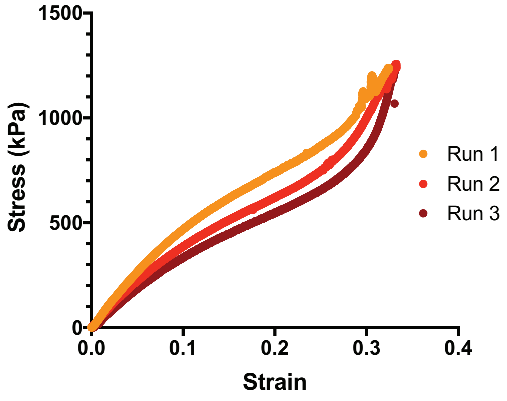

Supplementary Figure 10: Repeat stress-strain measurements of the same hydrogel-coated, anisotropic device. A degree of hysteresis was observed as cycle number increased indicative of plastic deformation of the 3D printed scaffold. The hydrogel coating remained intact throughout all three compression cycles.

Supplementary Table

Supplementary Table 1: Design parameters of the 3D printed scaffolds

Scaffold geometry	Scaffold material	Dimensions (cm)	Window size[#] (mm)	Pipe diameter (mm)
Mesh squares	Somos 9120	0.5 x 0.5<br>1 x 1<br>1.5 x 1.5<br>2 x 2	2.25 x 2.25	0.5
		1 x 1	2.25 x 2.25<br>5.5 x 5.5<br>8.75 x 8.75	0.5
		1 x 1	N.D.	0.25<br>0.5<br>1
Polyhedron	Somos 9120	2 x 2 (d x h)	N.D.	0.5
Pyramid	Stainless steel 17-4PH	4 x 2 (a x h)	N.D.	0.5
Tubular scaffold	AccuraClearVue	3 x 1 (l x d)	N.D.	0.5

a: base length; d: diameter; h: height; l: length; N.D.: not determined; X: window size was calculated as length x width for center-to-center distance between adjacent struts

Supplementary Methods

Modeling of suspended liquid films

In many applications, one aims to control the conformal surface coating of a porous or fenestrated template. For example, ceramic foams are often fabricated by coating an open-cell polymer foam with a slurry of ceramic particles. The ceramic is created by thermal decomposition of the polymer support followed by high temperature firing. The resultant structure should then be composed of ceramic struts that recapitulate the structure of the original polymer foam. However, attempts to fabricate thicker struts by increasing the slurry loading in the foam are only successful to a point. It becomes much more difficult to achieve a uniform distribution of the ceramic within the foam at increased volume fraction. In addition, the number of open cells that fill completely increases dramatically at relatively low loading.

This phenomenon is reminiscent of a process observed for the distribution of a liquid in a packed bed of spherical particles.[1] There is an inherent tendency for liquid to fill voids between particles rather than uniformly coat each particle as the volume fraction of liquid is increased in the porous body. An analogous phenomenon occurs during the wetting of a wire-mesh screen, such as a common window screen. Observations of wet screens reveal that the distribution of liquid in the mesh is such that only a thin film of water is on the wire of a given cell or the cell is completely filled. The distribution of the filled cells depends on the amount of water applied to the screen and no tendency toward liquid migration to form a more even distribution is observed.

Whether controlling strut thickness in porous ceramics, sintering metallic powders via liquid-phase sintering, or attempting to uniformly coat a mesh screen with a liquid, it is important to understand the underlying driving forces of the wetting process. Here, we consider a two-dimensional, square-cell mesh as a model system to model these observed phenomena.

Consider the case displayed in **Supplementary Figure 1a**. A small amount of liquid is placed on the mesh. The liquid distributes uniformly and segregates to the corners of each cell to minimize its total surface area. The radius of curvature at each of the liquid menisci is governed by the contact angle, θ, that the liquid makes with the solid. Increasing the volume fraction of liquid in the mesh causes the liquid-filled corners or fillets to grow. Eventually the fillets at adjacent corners touch and continued filling should result in the liquid distribution depicted in **Supplementary Figure 1b**. We refer to these modes as Phase I and Phase II filling, respectively, in the following calculations. Real experience with wet mesh screens demonstrates that Phase II filling is not observed physically. Rather, the liquid distribution becomes inhomogeneous as the volume fraction is increased and takes on the distribution shown in **Supplementary Figure 1c**, Inhomogeneous filling.

We show below that this behavior is a consequence of the surface energies of the liquid and solid in the system. The total Gibbs Energy of the system is minimized during an intermediate region between Phase I filling and Complete filling, in which there is a distribution of filled and unfilled cells. The necessary equations are derived below and solved to provide a quantitative assessment of the critical volume fraction for the occurrence of this phenomenon and the effect of the contact angle of the liquid. Importantly for our applications of completely coating a mesh scaffold, the total Gibbs Energy of the system is minimized at high saturation in the Complete filling case for a wide range of contact angles, $\theta < 90°$. Thus, a homogeneous suspended liquid film coating on arbitrary mesh scaffolds is physically feasible and energetically favorable for a wide range of materials.

Phase I filling

Phase I filling is defined as uniform liquid segregation to the corners and some exposed solid of the network. This is shown for an idealized, two-dimensional, square-cell mesh with cell length, ℓ, and total area of liquid filling in a cell, A_{L}, in **Supplementary Figure 1**. Consider the Gibbs Energy for a given cell, which is de-

termined by summing the products of the solid, liquid, and solid-liquid interface lengths with their respective surface energies.

$$\boxed{F_{\mathrm{I}} = 4\ell_{\mathrm{S}}\gamma_{\mathrm{S}} + 4\ell_{\mathrm{L}}\gamma_{\mathrm{L}} + 8\ell_{\mathrm{SL}}\gamma_{\mathrm{SL}}} \tag{1}$$

Note: the surface energies are related to the contact angle, θ, by Young's equation:

$$\gamma_{\mathrm{S}} = \gamma_{\mathrm{SL}} + \gamma_{\mathrm{L}} cos\theta \tag{2}$$

Here, ℓ_{S} is the length of exposed solid mesh, ℓ_{L} is the length of the exposed liquid, and ℓ_{SL} is the length of the solid-liquid interface. γ_{S} is the surface energy of the exposed solid, γ_{L} is the surface energy of the exposed liquid, and γ_{SL} is the surface energy of the solid-liquid interface. Therefore, in order to solve for the Gibbs Energy of Phase I filling, Supplementary Equation 1, we need to solve for ℓ_{S}, ℓ_{L}, and ℓ_{SL} in terms of A_{L} and θ using geometry.

Consider one corner (or fillet) of one cell from the two-dimensional square mesh, **Supplementary Figure 2b**. $\triangle ABE$ constitutes a right triangle as $\overline{BE}$ is tangent to the liquid arc at B. Therefore:

$$\boxed{\tan\frac{\phi}{2} = \frac{s}{r}} \tag{3}$$

Next, consider $\triangle BCE$. All angles must sum to π, therefore:

$$\begin{aligned} \pi &= \frac{\pi}{4} + \theta + x \\ x &= \frac{3\pi}{4} - \theta \end{aligned} \tag{4}$$

The Law of Sines provides, given $\sin\frac{\pi}{4} = \frac{\sqrt{2}}{2}$:

$$\begin{gathered} \frac{\ell_{\mathrm{SL}}}{\sin x} = \frac{s}{\sin\frac{\pi}{4}} \\ \boxed{\frac{\ell_{\mathrm{SL}}}{\sin\left(\frac{3\pi}{4} - \theta\right)} = \sqrt{2}\, s} \end{gathered} \tag{5}$$

Next, consider $\triangle ABC$. Here, the Law of Sines provides, given $\sin\frac{\pi}{4} = \frac{\sqrt{2}}{2}$ and that the angle subtending $\frown BD = \phi$:

$$\begin{gathered} \frac{\ell_{\mathrm{SL}}}{\sin\frac{\phi}{2}} = \frac{r}{\sin\frac{\pi}{4}} \\ \boxed{\frac{\ell_{\mathrm{SL}}}{\sin\frac{\phi}{2}} = \sqrt{2}\, r} \end{gathered} \tag{6}$$

In addition, the total area of liquid in a given cell, A_{L}, is four times the area of a single fillet, by symmetry. The area of a single fillet can be calculated from basic geometrical elements:

$$\frac{A_{\mathrm{L}}}{4} = \left[A_{\triangle BCD}\right] - \left[\left(A_{\triangledown ABD}\right) - \left(A_{\triangle ABD}\right)\right] \tag{7}$$

$$A_{\triangle BCD} = \frac{1}{2}\ell_{\mathrm{SL}}^2 \tag{8}$$

$$A_{\triangledown ABD} = \frac{1}{2}\phi r^2 \tag{9}$$

$$A_{\triangle ABD} = \frac{1}{2}r^2 \sin\phi \tag{10}$$

Therefore,

$$\boxed{\frac{A_{\mathrm{L}}}{4} = \frac{1}{2}\ell_{\mathrm{SL}}^2 - \frac{1}{2}\phi r^2 + \frac{1}{2}r^2 \sin\phi} \tag{11}$$

Supplementary Equations 3, 5, 6, and 11 comprise a set of equations that can be used to determine ℓ_{S}, ℓ_{L}, and ℓ_{SL}, given A_{L} and ℓ, the cell dimension. First solve Supplementary Equation 6 for r:

$$\boxed{r = \frac{\ell_{\mathrm{SL}}}{\sqrt{2}\sin\frac{\phi}{2}}} \tag{12}$$

and combine Supplementary Equation 12 with Supplementary Equation 3 to give

$$\begin{aligned}
\tan\frac{\phi}{2} &= \frac{\left(\sqrt{2}\sin\frac{\phi}{2}\right)s}{\ell_{\mathrm{SL}}} \\
\frac{\sin\frac{\phi}{2}}{\cos\frac{\phi}{2}} &= \frac{\left(\sqrt{2}\sin\frac{\phi}{2}\right)s}{\ell_{\mathrm{SL}}} \\
\frac{1}{\cos\frac{\phi}{2}} &= \frac{\sqrt{2}s}{\ell_{\mathrm{SL}}}
\end{aligned}$$

Solving for s gives:

$$s = \frac{\ell_{\mathrm{SL}}}{\sqrt{2}\cos\frac{\phi}{2}} \tag{13}$$

Supplementary Equation 13 can be combined with Supplementary Equation 5 to give

$$\frac{\ell_{\mathrm{SL}}}{\sin\left(\frac{3\pi}{4} - \theta\right)} = \sqrt{2}\left(\frac{\ell_{\mathrm{SL}}}{\sqrt{2}\cos\frac{\phi}{2}}\right)$$

or

$$\boxed{\sin\left(\frac{3\pi}{4} - \theta\right) = \cos\frac{\phi}{2}} \tag{14}$$

Supplementary Equation 14 describes the angle subtended by the liquid fillet, ϕ, given the contact angle, θ. Now, combine Supplementary Equation 12 with Supplementary Equation 7

$$\frac{A_L}{4} = \frac{1}{2}\ell_{SL}^2 - \frac{1}{2}\phi\left[\frac{\ell_{SL}}{\sqrt{2}\sin\frac{\phi}{2}}\right]^2 + \frac{1}{2}\left[\frac{\ell_{SL}}{\sqrt{2}\sin\frac{\phi}{2}}\right]^2 \sin\phi$$

$$= \frac{1}{2}\ell_{SL}^2 - \frac{1}{4}\phi\frac{\ell_{SL}^2}{\sin^2\frac{\phi}{2}} + \frac{1}{4}\sin\phi\frac{\ell_{SL}^2}{\sin^2\frac{\phi}{2}}$$

$$A_L = 2\ell_{SL}^2 - \phi\frac{\ell_{SL}^2}{\sin^2\frac{\phi}{2}} + \sin\phi\frac{\ell_{SL}^2}{\sin^2\frac{\phi}{2}}$$

$$= \ell_{SL}^2\left[2 + \frac{\sin\phi - \phi}{\sin^2\frac{\phi}{2}}\right] \quad (15)$$

Solving Supplementary Equation 15 for ℓ_{SL} gives

$$\boxed{\ell_{SL} = \sqrt{\frac{A_L}{2 + \frac{\sin\phi - \phi}{\sin^2\frac{\phi}{2}}}}} \quad (16)$$

Supplementary Equation 16 provides ℓ_{SL} given the fractional filling, A_L. NB: ϕ is calculated with Supplementary Equation 14.

The length of the liquid interface, ℓ_L, is given by

$$\ell_L = r\phi \quad (17)$$

Combining Supplementary Equation 17 and Supplementary Equation 12 gives

$$\boxed{\ell_L = \frac{\phi\ \ell_{SL}}{\sqrt{2}\sin\frac{\phi}{2}}} \quad (18)$$

Supplementary Equation 18 can be used with Supplementary Equations 16 and 14 to determine the length of the liquid interface, ℓ_L.

Finally, the length of the exposed solid interface, ℓ_S, can be obtained by inspection of **Supplementary Figure 2**. The length of one side of the cell, ℓ, is

$$\ell = \ell_S + 2\ell_{SL}$$

or

$$\boxed{\ell_S = \ell - 2\ell_{SL}} \quad (19)$$

Recall, that the Gibbs Energy of Phase I filling is calculated by multiplying the lengths for the solid, liquid, and solid-liquid interfaces by the appropriate surface energies. Thus, we can calculate the Gibbs Energy for a single cell given A_L. From **Supplementary Figure 2**, the appropriate sum is given by Supplementary Equation 1

$$\boxed{F_I = 4\ell_S\gamma_S + 4\ell_L\gamma_L + 8\ell_{SL}\gamma_{SL}}$$

Recall Young's Equation: $\gamma_S = \gamma_{SL} + \gamma_L\cos\theta$.

Phase II filling

The liquid distribution for the proposed Phase II filling completely coats the edges of each cell as shown in **Supplementary Figure 3**. The relationship between A_L and the appropriate lengths can be calculated by first considering the relation between r and A_L.

$$A_L = \ell^2 - \pi r^2$$

or

$$\boxed{r = \sqrt{\frac{\ell^2 - A_L}{\pi}}} \tag{20}$$

The length of the liquid interface, ℓ_L, is given by:

$$\ell_L = 2\pi r \tag{21}$$

Combining Supplementary Equation 21 with Supplementary Equation 20 gives

$$\boxed{\ell_L = 2\sqrt{\pi}\sqrt{\ell^2 - A_L}} \tag{22}$$

Therefore, the Gibbs Energy per cell for Phase II filling is

$$\boxed{F_{II} = 4\ell\gamma_{SL} + 2\gamma_L\sqrt{\pi}\sqrt{\ell^2 - A_L}} \tag{23}$$

Complete wetting: $\theta = 0°$

The case for complete wetting, where $\theta = 0°$, simplifies the analysis significantly. Supplementary Equation 14 becomes

$$\sin\frac{3\pi}{4} = \cos\frac{\phi}{2}$$

Therefore,

$$\boxed{\phi = \frac{\pi}{2}} \tag{24}$$

With $\phi = \frac{\pi}{2}$, Supplementary Equation 16 becomes

$$\begin{aligned}
\ell_{SL} &= \sqrt{\frac{A_{SL}}{2 + \frac{\sin\frac{\pi}{2} - \frac{\pi}{2}}{\sin^2\frac{\pi}{4}}}} \\
&= \sqrt{\frac{A_{SL}}{2 + \frac{1 - \frac{\pi}{2}}{\left(\frac{\sqrt{2}}{2}\right)^2}}} \\
&= \sqrt{\frac{A_{SL}}{2 + 2\left(1 - \frac{\pi}{2}\right)}} \\
&= \sqrt{\frac{A_{SL}}{4 - \pi}}
\end{aligned}$$

Therefore,

$$\boxed{\ell_{\mathrm{SL}} = \sqrt{\frac{A_{\mathrm{SL}}}{4-\pi}}} \tag{25}$$

With $\phi = \frac{\pi}{2}$, Supplementary Equation 18 becomes

$$\begin{aligned}
\ell_{\mathrm{L}} &= \frac{\frac{\pi}{2}\,\ell_{\mathrm{SL}}}{\sqrt{2}\sin\frac{\pi}{4}} \\
&= \frac{\frac{\pi}{2}\,\ell_{\mathrm{SL}}}{\sqrt{2}\frac{\sqrt{2}}{2}} \\
&= \frac{\pi}{2}\,\ell_{\mathrm{SL}}
\end{aligned} \tag{26}$$

which when combined with Supplementary Equation 25 gives

$$\boxed{\ell_{\mathrm{L}} = \frac{\pi}{2}\sqrt{\frac{A_{\mathrm{L}}}{4-\pi}}} \tag{27}$$

Finally, Supplementary Equation 19 becomes

$$\boxed{\ell_{\mathrm{S}} = \ell - 2\sqrt{\frac{A_{\mathrm{L}}}{4-\pi}}} \tag{28}$$

Therefore, the Gibbs Energy for Phase I filling becomes

$$\begin{aligned}
F_{\mathrm{I}} &= 4\gamma_{\mathrm{S}}\left[\ell - 2\sqrt{\frac{A_{\mathrm{L}}}{4-\pi}}\right] + 4\gamma_{\mathrm{L}}\frac{\pi}{2}\sqrt{\frac{A_{\mathrm{L}}}{4-\pi}} + 8\gamma_{\mathrm{SL}}\frac{\pi}{2}\sqrt{\frac{A_{\mathrm{L}}}{4-\pi}} \\
&= 4\gamma_{\mathrm{S}}\ell - 8\gamma_{\mathrm{S}}\sqrt{\frac{A_{\mathrm{L}}}{4-\pi}} + 2\pi\gamma_{\mathrm{L}}\sqrt{\frac{A_{\mathrm{L}}}{4-\pi}} + 8\gamma_{\mathrm{SL}}\sqrt{\frac{A_{\mathrm{L}}}{4-\pi}} \\
&= 4\gamma_{\mathrm{S}}\ell + \left[2\pi\gamma_{\mathrm{L}} + 8\gamma_{\mathrm{SL}} - 8\gamma_{\mathrm{S}}\right]\sqrt{\frac{A_{\mathrm{L}}}{4-\pi}}
\end{aligned}$$

The surface energies are related through Young's Equation, $\gamma_{\mathrm{S}} = \gamma_{\mathrm{SL}} + \gamma_{\mathrm{L}}\cos\theta$, which for $\theta = 0°$ gives $\gamma_{\mathrm{S}} = \gamma_{\mathrm{SL}} + \gamma_{\mathrm{L}}$.

Therefore,

$$\begin{aligned}
F_{\mathrm{I}} &= 4\left(\gamma_{\mathrm{SL}} + \gamma_{\mathrm{L}}\right)\ell + \left[2\pi\gamma_{\mathrm{L}} + 8\gamma_{\mathrm{SL}} - 8\left(\gamma_{\mathrm{SL}} + \gamma_{\mathrm{L}}\right)\right]\sqrt{\frac{A_{\mathrm{L}}}{4-\pi}} \\
&= 4\left(\gamma_{\mathrm{SL}} + \gamma_{\mathrm{L}}\right)\ell + \left(2\pi\gamma_{\mathrm{L}} - 8\gamma_{\mathrm{L}}\right)\sqrt{\frac{A_{\mathrm{L}}}{4-\pi}}
\end{aligned}$$

which, when reformulated gives

$$\boxed{F_{\mathrm{I}} = 4\left(\gamma_{\mathrm{SL}} + \gamma_{\mathrm{L}}\right)\ell - (8 - 2\pi)\,\gamma_{\mathrm{L}}\sqrt{\frac{A_{\mathrm{L}}}{4-\pi}}} \tag{29}$$

Upon inspection, Supplementary Equation 29 implies that F_{I} decreases as A_{L} increases, which should be the case if the liquid favorably wets the solid as with $\theta = 0°$.

We then consider a reduced Gibbs Energy per cell, $\frac{F_{\mathrm{I}}}{\gamma_{\mathrm{L}}\ell}$, which can be thought of as similar to the Gibbs Energy per volume scaled by the surface energy of the liquid. For the case where $\theta = 0°$:

$$\boxed{\frac{F_{\mathrm{I}}}{\gamma_{\mathrm{L}}\ell} = 4\left(1 + \frac{\gamma_{\mathrm{SL}}}{\gamma_{\mathrm{L}}}\right) - (8 - 2\pi)\sqrt{\frac{a_{\mathrm{L}}}{4-\pi}}} \tag{30}$$

where $a_{\mathrm{L}} = \frac{A_{\mathrm{L}}}{\ell^2}$ and is the saturation of the mesh.

A similar transformation can be performed for Phase II filling:

$$\boxed{\frac{F_{\mathrm{II}}}{\gamma_{\mathrm{L}}\ell} = 4\frac{\gamma_{\mathrm{SL}}}{\gamma_{\mathrm{L}}} + 2\sqrt{\pi}\sqrt{1 - a_{\mathrm{L}}}} \tag{31}$$

Supplementary Equation 30 is only applicable when

$$\ell_{\mathrm{SL}} < \frac{\ell}{2} \tag{32}$$

since Phase II filling begins at larger values of a_{L}. Combining Supplementary Equation 32 with Supplementary Equation 25 gives the limiting saturation for Phase I filling, $a_{\mathrm{L_c}}$.

$$\sqrt{\frac{A_{\mathrm{L}}}{4-\pi}} < \frac{\ell}{2}$$
$$\frac{A_{\mathrm{L}}}{4-\pi} < \frac{\ell^2}{4}$$
$$\frac{A_{\mathrm{L}}}{\ell^2} < \frac{4-\pi}{4}$$
$$a_{\mathrm{L}} < 1 - \frac{\pi}{4}$$

Therefore, with $\theta = 0°$

$$\boxed{a_{\mathrm{L_c}} = 1 - \frac{\pi}{4} \approx 0.215} \tag{33}$$

Partial wetting: $\theta > 0°$

The critical equations, as described above, are:

$$\phi = 2\arccos\left[\sin\left(\frac{3\pi}{4} - \theta\right)\right] \tag{34}$$

$$\ell_{\mathrm{SL}} = \sqrt{\frac{A_{\mathrm{L}}}{2 + \frac{\sin\phi - \phi}{\sin^2\frac{\phi}{2}}}} \tag{35}$$

$$\ell_{\mathrm{L}} = \frac{\phi\,\ell_{\mathrm{SL}}}{\sqrt{2}\sin\frac{\phi}{2}} \tag{36}$$

$$\ell_{\mathrm{S}} = \ell - 2\ell_{\mathrm{SL}} \tag{37}$$

$$F_{\mathrm{I}} = 4\ell_{\mathrm{S}}\left(\gamma_{\mathrm{SL}} + \gamma_{\mathrm{L}}\cos\theta\right) + 4\ell_{\mathrm{L}}\gamma_{\mathrm{L}} + 8\ell_{\mathrm{SL}}\gamma_{\mathrm{SL}} \tag{38}$$

$$F_{\mathrm{II}} = 4\ell\gamma_{\mathrm{SL}} + 2\gamma_{\mathrm{L}}\sqrt{\pi}\sqrt{\ell^2 - A_{\mathrm{L}}} \tag{39}$$

As above, Phase I filling can only occur when $\ell_{\mathrm{SL}} < \frac{\ell}{2}$. This criterion depends directly on the wetting angle, such that

$$\sqrt{\frac{A_{\mathrm{L}}}{2 + \frac{\sin\phi - \phi}{\sin^2\frac{\phi}{2}}}} < \frac{\ell}{2} \tag{40}$$

where $\phi = 2\arccos\left[\sin\left(\frac{3\pi}{4} - \theta\right)\right]$.

Rewriting Supplementary Equation 40 with the saturation of the network, $a_{\mathrm{L}} = \frac{A_{\mathrm{L}}}{\ell^2}$, gives

$$\sqrt{\frac{a_{\mathrm{L}}}{2 + \frac{\sin\phi - \phi}{\sin^2\frac{\phi}{2}}}} < \frac{1}{2}$$

$$\frac{a_{\mathrm{L}}}{2 + \frac{\sin\phi - \phi}{\sin^2\frac{\phi}{2}}} < \frac{1}{4}$$

therefore

$$\boxed{a_{\mathrm{L}} < \frac{1}{2} + \frac{\sin\phi - \phi}{4\sin^2\frac{\phi}{2}} \text{ where } \phi = 2\arccos\left[\sin\left(\frac{3\pi}{4} - \theta\right)\right]} \tag{41}$$

Finally, the full set of reduced equations for a partially wetting liquid are:

$$\phi = 2\arccos\left[\sin\left(\frac{3\pi}{4} - \theta\right)\right] \tag{42}$$

$$\frac{\ell_{\text{SL}}}{\ell} = \sqrt{\frac{a_{\text{L}}}{2 + \frac{\sin\phi - \phi}{\sin^2\frac{\phi}{2}}}} \tag{43}$$

$$\frac{\ell_{\text{L}}}{\ell} = \frac{\phi\frac{\ell_{\text{SL}}}{\ell}}{\sqrt{2}\sin\frac{\phi}{2}} \tag{44}$$

$$\frac{\ell_{\text{S}}}{\ell} = 1 - 2\frac{\ell_{\text{SL}}}{\ell} \tag{45}$$

$$a_{\text{L}}^{\text{I}} = \frac{1}{2} + \frac{\sin\phi - \phi}{4\sin^2\frac{\phi}{2}} \tag{46}$$

$$\boxed{\frac{F_{\text{I}}}{\gamma_{\text{L}}\ell} = 4\frac{\ell_{\text{S}}}{\ell}\left(\frac{\gamma_{\text{SL}}}{\gamma_{\text{L}}} + \cos\theta\right) + 4\frac{\ell_{\text{L}}}{\ell} + 8\frac{\ell_{\text{SL}}}{\ell}\frac{\gamma_{\text{SL}}}{\gamma_{\text{L}}}} \tag{47}$$

$$\boxed{\frac{F_{\text{II}}}{\gamma_{\text{L}}}\ell = 4\frac{\gamma_{\text{SL}}}{\gamma_{\text{L}}} + 2\sqrt{\pi}\sqrt{1 - a_{\text{L}}}} \tag{48}$$

Supplementary Discussion

The above derivation relates the Gibbs Energy of a liquid-coated, two-dimensional mesh to the saturation of the network (a_{L}), given the contact angle (θ) of the liquid. By inspecting the geometry, the lengths of solid-vapor (solid, ℓ_{S}), liquid-vapor (liquid, ℓ_{L}), and solid-liquid (ℓ_{SL}) contact can be calculated for a given saturation. The sum of the products of these lengths and the respective surface energies provides the Gibbs Energy, which was normalized for the purpose of this analysis by dividing by the product of the liquid surface energy (γ_{L}) and the dimension of a cell in the mesh (ℓ). The reduced Gibbs Energy as a function of saturation is shown in **Supplementary Figure 4** for a liquid with $\theta = 0°$.

There are two clear observations. First, the absolute value of the Gibbs Energy depends on the ratio of the solid-liquid and liquid surface energies; however, the general features and physical implications do not change with this ratio. Therefore, $\frac{\gamma_{\text{SL}}}{\gamma_{\text{L}}}$ is set to unity for the purposes of this discussion. Second, the reduced Gibbs Energy displays a change in character as the saturation increases, with a change in sign in the second derivate of the reduced Gibbs Energy. At very low saturation, the reduced Gibbs Energy is concave up. This is caused by the high surface-to-volume ratio for the liquid fillets for low saturation and, thus, the reduced Gibbs Energy decreases with additional liquid. The surface-to-volume ratio decreases as the fillet grows indicating that the decrease in reduced Gibbs Energy is not as drastic with further increases in saturation. This causes the concave up behavior in the low saturation regime or Phase I filling. In contrast, during Phase II filling, the surface area-to-volume ratio changes rapidly with saturation only when the cell is nearly full. This is mechanical in nature - a small pore in the center of the cell will decrease in size quickly only when the cell is near complete saturation - and does not depend on θ. Therefore, the reduced Gibbs Energy of Phase II filling is concave down. For $\theta = 0°$ the transition from Phase I filling to Phase II filling occurs at $a_{\text{L}_\text{c}} \approx 0.215$.

The observed change in sign in the second derivative of the reduced Gibbs Energy indicates that a phase separation occurs. This corresponds to the physical situation of Inhomogeneous filling of the mesh, whereby some wells are filled completely and the remaining cells contain liquid only in the fillets. The composition of the two phases is determined by the intersection of the tangent line shown in **Supplementary Figure 5**. The high

saturation phase corresponds to completely filled cells and the lower saturation phases corresponds to cells with $a_L \approx 0.052$ for $\theta = 0°$. The mechanically-driven phase separation occurs as the reduced Gibbs Energy for the Phase II filling regime is concave down and liquid will redistribute to close the pores in cells that have entered Phase II filling. The analysis highlights that Inhomogeneous filling occurs at relatively low saturation and additional liquid creates more completely filled cells rather than increasing the volume of fillets or cells that exist in Phase II filling. While this phenomenon is problematic for conformal surface coating in ceramic foams, it is beneficial for creating mesh scaffolds that are completely coated with suspended liquid films. By driving the saturation $a_L \to 1$ with an immersive dip coating, one can easily access the energetically favorable Complete filling regime.

The above analysis has considered $\theta = 0°$. The effect of contact angle on the reduced Gibbs Energy as a function of saturation is shown in **Supplementary Figure 6**. For $\theta = [0°, 45°)$, the above conclusions hold. Complete wetting is more energetically favorable than fillet filling. However, based on the assumptions of this analysis, Phase I filling will not occur for $\theta \geq 45°$ and in this regime the device will either be completely coated or not coated at all. Further, based on the assumptions of this analysis, the complete wetting case is no longer more energetically favorable for $\theta \geq 90°$ indicating that Complete filling will not occur. This shows that, while complete wetting of reticulated scaffolds is possible and favorable, it requires a liquid precursor solution that is sufficiently wetting on the selected scaffold material.

Supplementary References

[1] Shaw, T. M. Liquid redistribution during liquid-phase sintering. *J. Am. Ceram. Soc.* **69**, 27–34 (1986).
